# Cognitive impairment in patients with Neuro‐Sjögren

**DOI:** 10.1002/acn3.51123

**Published:** 2020-07-06

**Authors:** Tabea Seeliger, Lena Jacobsen, Merle Hendel, Lena Bönig, Nils K. Kristian Prenzler, Thea Thiele, Diana Ernst, Torsten Witte, Martin Stangel, Bruno Kopp, Thomas Skripuletz

**Affiliations:** ^1^ Department of Neurology Hanover Medical School Hanover Germany; ^2^ Department of Otolaryngology Hanover Medical School Hanover Germany; ^3^ Department of Clinical Immunology and Rheumatology Hanover Medical School Hanover Germany

## Abstract

**Objective:**

Extraglandular neurological manifestations of Sjögren’s syndrome are increasingly recognized, defining the disease entity of Neuro‐Sjögren. Neuropsychological assessment of patients with Sjögren’s syndrome has hitherto been performed on predominantly rheumatological cohorts. These studies revealed a wide variety of prevalence rates for cognitive impairment (22–80%), while variable cut‐off criteria for detection of cognitive impairment were applied. Attentional functions have not yet been thoroughly investigated in these patients, although they clearly represent relevant aspects of cognitive functioning in daily life.

**Methods:**

We therefore conducted extensive neuropsychological assessment based on two neuropsychological test batteries [i.e., the extended German version of the Consortium to Establish a Registry for Alzheimer’s Disease Neuropsychological Assessment Battery (CERAD‐PLUS), and the test battery for attentional performance (TAP) as a well‐established assessment of attentional functions in the German‐speaking part of Europe].

**Results:**

Sixty‐four patients with Neuro‐Sjögren, who were treated at our university hospital between December 2016 and January 2019, were included. Evidence for the presence of cognitive impairment was found in 55% of patients with Neuro‐Sjögren. The degree of cognitive impairment ranged from mild (38%) to severe (17%). Attentional and mnemonic subtests showed pronounced cognitive impairment in patients with Neuro‐Sjögren.

**Interpretation:**

Our results suggest that a substantial proportion of patients with Neuro‐Sjögren suffer from cognitive impairment, putatively as a corollary of attentional deficits, which might exert adverse effects on occupational abilities, other cognitive functions, and social role functioning.

## Introduction

Sjögren’s syndrome is an autoimmune disease with lymphocytic infiltration and consecutive inflammatory destruction of salivary and lacrimal glands causing the typical sicca symptoms in eyes and mouth.^1^, ^2^ Extraglandular neurological manifestations of Sjögren’s syndrome are increasingly recognized, forming the entity of Neuro‐Sjögren.^3^, ^4^, ^5^, ^6^, ^7^ While peripheral neuropathy and cranial nerve affection^4^, ^5^ are the most prevalent neurological symptoms of Sjögren’s syndrome with rates of up to 60%, there have been several studies suggesting the involvement of the central nervous system through spinal cord involvement, headache,^8^ and cognitive dysfunction.^9^, ^10^, ^11^, ^12^ Neuropsychological testing in patients with Sjögren’s syndrome has hitherto been performed on mostly rheumatological cohorts with the typical female preponderance.^13^ Furthermore, investigation of attentional function has not yet been thoroughly analyzed in patients with Sjögren’s syndrome, although it represents one of the most relevant aspects of cognitive functionality in daily life and its impairment leads to disposition of neuropsychiatric comorbidity.^14^ We therefore conducted this study to determine the prevalence and character of cognitive dysfunction and attention in patients with Neuro‐Sjögren in accordance with the current classification criteria for Sjögren’s syndrome.^15^

## Methods

### Patient selection

Patients were recruited from inpatients of the neurological department of the Hanover Medical School, who were treated between December 2016 and January 2019. Inclusion was obtained if patients fulfilled the current classification criteria for Sjögren’s syndrome according to the American College of Rheumatology and the European League Against Rheumatology^15^ and if neuropathy was clinically and electrophysiologically and/or histopathologically (in the sense of a proven small fiber neuropathy) evident. For adequate diagnostics, Anti‐SSA(Ro)‐ and Anti‐SSB(La)‐antibody testing as well as a minor salivary gland biopsy were performed. Objective xerophthalmia and xerostomia were assessed via Schirmer test (lacrimal fluid < 5 mm/5 min in at least one eye) and Saxon test (saliva production of <3.5 g/2 min). Exclusion criteria were diminished speech comprehension, remarkable vision or hearing loss, and relevant functional deficits in the upper limb movement, as those represented crucial skills for valid neuropsychological testing. Patients with major depression or other psychiatric disorder, prior learning disorder, multiple sclerosis, alcohol abuse, carcinoma, hypothyroidism, and vitamin B12 deficiency were also not tested. Seven patients suffered from hypothyroidism but were substituted to a euthyroid metabolic status at the time of testing. Four patients were previously diagnosed with vitamin B12 deficiency but were adequately substituted at the time of testing.

### Neuropsychological testing

Neuropsychological testing was performed by the “Consortium in order to establish a registry for Alzheimer's Disease” (CERAD‐PLUS)^16^ and the “Test battery for attentional performance” (TAP).^17^ We followed a fully standardized approach in which the patients attended the established neuropsychological tests and their performance was compared to large samples of normative data. Therefore, a control group was not included. The subtest results were collected as z‐values, respectively, in relation to a previously established large‐sized norm sample and therefore corrected for age, gender, and education. The time of scholar education was therefore inquired, independent of attended school modality.

The CERAD‐Plus test and the TAP test were both selected because large standardization data sets are available (see the sample size information in Table 3). As a result, patient’s performance can be expressed in *z* scores (M = 0, SD = 1). *Z* scores below 0 indicate that the group’s performance is impaired compared to that of the respective age‐, education‐, and gender‐matched large‐sized standardization control sample.

#### “Consortium in order to establish a registry for Alzheimer's Disease” (CERAD‐PLUS)

The CERAD‐PLUS is an established cognitive test battery originally developed as a screening for Alzheimer´s disease and designed to test executive functions, visuospatial understanding, language, and memory function through 12 subtests.^16^, ^18^, ^19^ Table 1 displays a short description of the applied subtests following our previously published methodology.^20^

**Table 1:** Short description of the applied CERAD‐PLUS subtests.

CERAD‐PLUS subtest	Subtest task
Semantic fluency	Number of listed animals in 60 sec
Boston naming test	Number of correctly labeled items (e.g., a house, a camel, a volcano, a tree) (out of 15)
Wordlist learning	Immediate reproduction of 10 previously provided terms (threefold repetition)
Wordlist learning recall	Delayed reproduction of the 10 previously provided terms after completing an interim task
Wordlist learning intrusions	Number of falsely reproduced terms on Wordlist learning and Wordlist learning recall
Wordlist learning recognition	Identification of the previously provided terms among 10 distractor words
Figures drawing	Reproduction of provided figures (including a circle, a cube, a rhombus, and a configuration of rectangles) next to the original
Phonemic fluency	Number of listed words starting with the letter “S” in 60s
Figures recall	Delayed reproduction of the previously provided figures (including a circle, a cube, a rhombus, and a configuration of rectangles) after an interim task
Trail making test A	Connection of distributed numbers in numerical order (measured in seconds)
Trail making test B	Interchanging connection of numbers and letters in numerical and alphabetical order (from 1 to A to 2 to B etc.) (measured in seconds)

#### “Test battery for attentional performance” (TAP)

Four subtests of the TAP were used to assess attentional performance.^21^, ^22^

##### Alertness

This subtest measured the participants’ simple reaction time. Visual stimuli (a cross appearing on a monitor) were presented either alone (condition A = visual stimulus) or preceded by a warning sound (condition B = visual stimulus with a warning sound). Stimuli were presented in a fully automated ABBA blocked trials pattern. Intervals between warning sound (if provided) and visual stimulus were randomly chosen. Twenty visual stimuli were presented to the participants (test duration approximately 5 min).

##### Divided attention

In this dual task, participants had to simultaneously observe visual and auditory distractor stimuli and were asked to identify two previously specified stimuli: 1. (visual) a square, which was formed by smaller crosses and 2. (auditory) a tone repetition in a series of alternating high and low pitched tones. In total, 100 visual and 200 auditory stimuli were presented (test duration approximately 5 min).

##### Working memory

Testing working memory required a constant flow of information through participants’ short‐term memory. This was achieved by a continuously presented sequence of numbers with numbers appearing one at a time. The participants had to recognize when a number equaled the penultimate (which was no longer visible and therefore had to be kept in mind). About 100 numbers were provided, while 15 represented a relevant stimulus (test duration approximately 5 min).

##### Sustained attention

Sustained attention was tested through visual presentation of a sequence of colored shapes. Participants had to recognize whether two consecutively presented figures matched in shape or color. About 450 stimuli were presented with 54 stimuli representing a relevant stimulus (test duration 15 min).

For the subtests of divided attention, working memory, and sustained attention, the number of false positive errors was protocolled.

### Data analysis and interpretation

All data were processed as *z*‐values through comparison to a standard sample and were therefore corrected for age, education, and gender. Subtest results ≤−1.5 standard deviation (SD) and >−2.5 SD were classified as mild impairment and results ≤−2.5 SD as severe impairment. Overall cognitive impairment was diagnosed if ≥3 subtest results showed mild reduction of cognitive function or if ≥2 subtests showed reduction of cognitive function with at least one of them severe. The degree of overall cognitive impairment was then classified in the following manner:

Severe overall impairment: reduction of cognitive function in ≥2 tests with at least one of them severe.

Mild overall impairment: mild reduction of cognitive function in ≥3 tests.

### Statistical analysis and data collection

Statistical data analysis was obtained using SPSS V24 (IBM, USA 2017). Testing for normal distribution was performed by Shapiro–Wilk. Parametric data were tested using one sample *t*‐tests, and nonparametric data were analyzed by Wilcoxon tests. Correlation analysis for nonparametrical, ordinal data was performed via Kruskal–Wallis test. The confounding factor analysis was performed via Chi‐square test. Data were collected and documented anonymously. Missing values were descriptively handled.

### Ethical approval

Approval of the study by the local ethics committee of the Hanover Medical School was obtained (No. 8270_BO_S_2019). All patients provided their informed consent.

## Results

### Patient’s characteristics

Sixty‐four patients were included in the analysis, who fulfilled the current classification criteria for Sjögren’s syndrome and showed electrophysiological and/or histopathological evidence of neuropathy and/or were diagnosed with myelitis. 45/64 patients were hospitalized during testing. Hospital admission was due to routine diagnostics and/or therapy of chronic symptoms without acute deterioration in 40/45 patients. 5/45 were hospitalized for evaluation of acute symptom onset. Baseline characteristics are fully displayed in Table 2. Patients showed a median age at the time of testing of 59 years (range 32–84 years) and 63% were female. Objective xerophthalmia was found in 55 patients (86%) and objective xerostomia in 30 patients (47%). Forty‐eight patients showed a definite histopathologic sialadenitis of either grade 3 (1 focus of a lymphocytic infiltration per 4 mm of salivary tissue) or grade 4 (>1 focus of a lymphocytic infiltration per 4 mm of salivary tissue) according to Chisholm and Mason.^23^ Twenty‐eight patients (44%) were seropositive for anti‐SSA(Ro) antibodies and six patients (9%) for anti‐SSB(La) antibodies. Determination of the classification criteria score^15^ via summation of applicable items for every patient [evident xerostomia (1 point), xerophthalmia (1 point), definite sialadenitis (3 points), and/ or anti‐SSA(Ro) antibody‐positivity (3 points)] lead to a mean score of 4.9 points. Neurological manifestation presented as affection of the peripheral nervous system in 60/64 patients and as inflammatory myelitis in 6/64 patients (aquaporin‐4‐antibody positive in 2/6), while both entities overlapped in two of those patients. Twenty‐eight patients showed a primarily demyelinating neuropathy, while in nine, a primarily axonal neuropathy was found. Sixteen patients suffered from a mixed axonal and demyelinating neuropathy. In seven patients, biopsy revealed a small fiber neuropathy. Painful sensory neuropathy was evident in 35/64 patients. Pain‐related medications were taken by 17/64 patients (gabapentin in six cases, pregabalin in eight cases, amitriptyline in one case, and opioids in two cases). Even if this was no explicit focus of this investigation, 26/64 patients had undergone magnetic resonance imaging (MRI) of the brain during routine diagnostics. 6/26 patients with available cerebral MRI showed T2‐hyperintensities indicating a microangiopathic pattern in frontoparietal subcortical regions. The other 20 brain MRIs did not reveal a pathology.

**Table 2:** Baseline parameters of included patients.

*n* (total)	64		
Female gender	40 (63%)		
Objective xerophthalmia, *n* (%)	55 (86%)		
Objective xerostomia, *n* (%)	30 (47%)		
Anti‐SSA(Ro) antibodies positive, *n* (%)	28 (44%)		
Anti‐SSB(La) antibodies positive, *n* (%)	6 (9%)		
Minor salivary gland biopsy positive, *n* (%)	48 (75%)		
Diagnostic score, mean	4.9		

	All	Female	Male
Age [in years] at time of testing, median (range)	59 (32–84)	54 (33–84)	70 (50–83)
Years of education, median (range)	13 (8–18)	13 (8–18)	12 (8–17)

### Cognitive impairment

**Figure 1:**
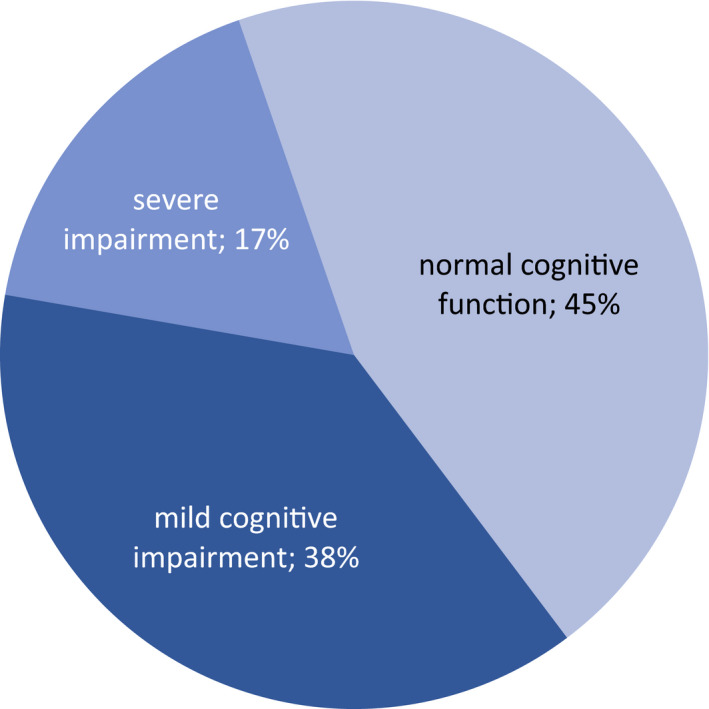
Severity of cognitive impairment in patients with Neuro‐Sjögren.

Data analysis revealed cognitive impairment in 35/64 patients (55%). The degree of cognitive impairment was severe in 11/64 patients (17%) and mild in 24/64 patients (38%) (Figure 1). The majority of the TAP subtests and additionally the CERAD‐PLUS subtests of Semantic Fluency, Wordlist Learning, Wordlist Learning Recall, and Wordlist Learning Recognition showed significant cognitive impairment in patients with Neuro‐Sjögren (Table 3). As there were some missing values due to apparent loop holes in standardization values, technical issues, or due to patients unable to complete the given task, tests were always interpreted in relation to the number of completed tests (detailed in Table 3). The TAP subtest of sustained attention provided the most missing values as the software often did not provide standardized values. Additionally, the TAP subtest of sustained attention was most difficult for patients to perform, resulting in 46/64 complete subtest results (72%), while the remaining TAP subtests were completed by 60–64/64 patients (94–100%).

**Table 3:** Subtest results. Green highlighting indicates significantly reduced performance compared to the norm sample.

	Size of standardization sample	Number of valid results	Median	SD	*P*‐value
CERAD‐PLUS subtests					
Semantic fluency	1100^#^	63 (98%)	−0.48	1.13	0.001^1^
Boston naming test	1100^#^	63 (98%)	0.39	0.98	0.113^2^
Wordlist learning	1100^#^	63 (98%)	−0.64	1.33	0.000^1^
Wordlist learning recall	1100^#^	63 (98%)	−0.51	1.00	0.001^1^
Wordlist learning intrusions	1100^#^	63 (98%)	0.33	1.06	0.470^2^
Wordlist learning recognition	1100^#^	63 (98%)	−0.50	0.96	0.000^1^
Figures drawing	1100^#^	63 (98%)	0.52	1.05	0.349^2^
Figures recall	1100^#^	63 (98%)	−0.27	1.26	0.609^2^
Phonemic fluency	604^#^	63 (98%)	−0.22	1.02	0.002^1^
Trail making test A	604^#^	63 (98%)	0.04	1.39	0.569^1^
Trail making test B	604^#^	59 (92%)	−0.14	1.49	0.735^1^
TAP subtests					
Alertness without warning sound (reaction time)	604^##^	64 (100%)	−0.99	1.01	0.000^2^
Alertness with warning sound (reaction time)	604^##^	64 (100%)	−0.92	0.95	0.000^2^
Divided attention false positive (errors, *N*)	808^##^	63 (98%)	−0.71	1.01	0.000^1^
Working memory false positive (errors, *N*)	322^##^	60 (94%)	−0.31	0.96	0.001^2^
Sustained attention total false positive (errors, *N*)	188^##^	46 (72%)	0.41	1.21	0.046^2^

References: ^#^ https://www.memoryclinic.ch/de/main‐navigation/neuropsychologen/cerad‐plus/auswertungprogramme/cerad‐plus‐online/. ^##^ https://www.psytest.net/index.php?page=normierung&hl=de_DE

^1^ Analysis via two‐sided *t*‐test.

^2^ Analysis via Wilcoxon test.

**Figure 2:**
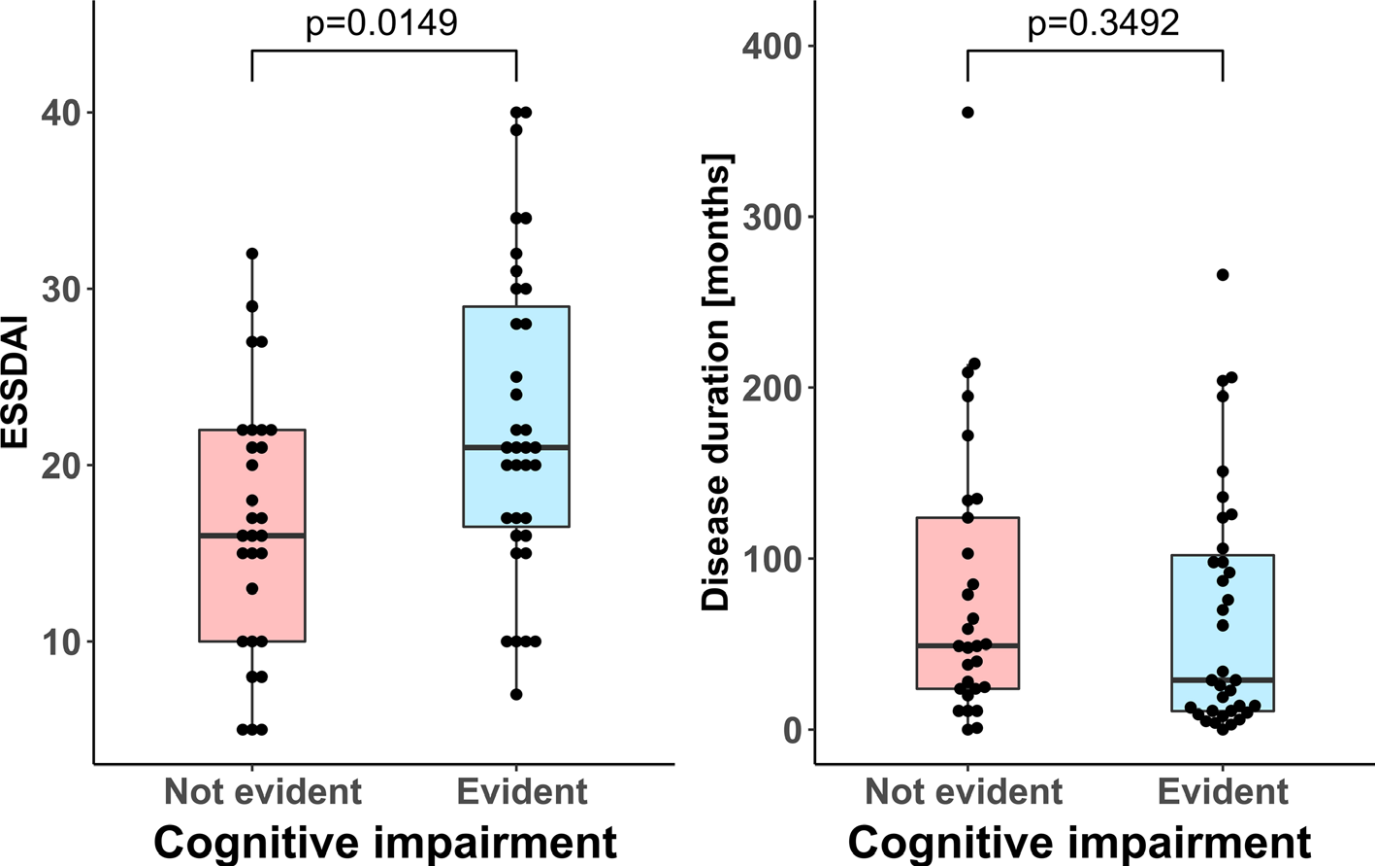
Correlation between disease activity, disease duration, and presentation of cognitive impairment (via Kruskal–Wallis test).

Disease activity was investigated via the EULAR Sjögren's syndrome disease activity index (ESSDAI) and showed a significant correlation with the presentation of cognitive impairment (*P* = 0.0149). However, disease duration did not correlate with the presentation of cognitive impairment (*P* = 0.3492). The analysis is illustrated in Figure 2.

### Potential confounding factors

Potential confounding factors were analyzed. As shown in Table 4, neuropathic pain, pain‐related medication, and hospitalization did not significantly confound the presence of cognitive impairment.

**Table 4:** Subgroup analysis comparing cognitive impairment in accordance with potential confounding factors.

Potential confounder	Cognitive impairment evident	Cognitive impairment not evident	*P*‐value
Neuropathic pain	16/35 (46%)	19/35 (54%)	0.1131
No neuropathic pain	19/29 (66%)	10/29 (34%)	
Pain‐related medication	9/17 (53%)	8/17 (47%)	0.8660
No pain‐related medication	26/47 (55%)	21/47 (44%)	
Hospitalization	28/45 (62%)	17/45 (38%)	0.0623
No hospitalization	7/19 (37%)	12/19 (63%)	
Admission chronic	25/40 (63.5%)	15/40 (37.5%)	0.9134
Admission acute	3/5 (60%)	2/5 (40%)	

## Discussion

### Cognitive impairment in patients with Neuro‐Sjögren

Our data show cognitive impairment (norm sample corrected) for 55% of patients with Neuro‐Sjögren independent of subjective complaints. The severity ranged from severe in 17% to mild in 38% of patients.

This is a high rate of cognitive dysfunction, but is also generally in accordance with rates previously reported for patients with Sjögren’s syndrome without neurological focus (ranging from 22 to 80%).^24^, ^25^ For patients with certain additional conditions – for example, small fiber neuropathy with chronic pain syndrome – even higher rates have been proposed.^26^ However, most of the previous studies investigated small cohorts with sizes between 10 and 39.^9^, ^11^, ^12^, ^13^, ^24^, ^25^, ^27^, ^28^, ^29^ Our cohort included 64 patients, which is a comparatively large number for a rare disease. More importantly, the threshold definition of cognitive impairment was often lower in comparison to our applied definition, which results in a more robust testing on our part, antagonizing overestimation.^10^ Finally, in‐between‐study‐comparability is diminished, as the applied methodology shows a wide variety. Especially, extensive attentional performance testing has not yet been performed as extensively as in our study, with different subtests for alertness, divided attention, working memory, and sustained attention.

Interestingly, attentional impairment was evident on almost all TAP subtests, although attentional functions have not yet been described as predominantly reduced. This effect is probably a result of the applied extensive testing in our study and shows that potential attentional performance deficits in patients with Sjögren’s syndrome have hitherto been underestimated. Previously described deficits included mainly memory functions^30^, ^31^ or visuospatial/executive functions,^29^ which were also significantly impaired in our cohort (Semantic and Phonemic Fluency and Wordlist Learning Subtests).

### Gender distribution

There was a comparatively high number of male participants (37.5%) in our cohort, although Sjögren’s syndrome patients are established to show a female preponderance. This, however, aligns with our previously published cohort of patients with Sjögren’s syndrome and severe neuropathy with a 1:1 female/male ratio. The high fraction of male patients further supports the hypothesis that Neuro‐Sjögren patients present with distinct features in comparison to predominantly rheumatological cohorts of patients with Sjögren’s syndrome.^3^

### Cognitive impairment – confounding factor or direct disease effect?

It remains notional, whether the reported high rate of cognitive impairment in patients with Neuro‐Sjögren (i.e., Sjögren’s syndrome combined with neurological extraglandular manifestations) is a result of confounding factors, such as disease‐associated chronic pain syndrome, latent metabolic or hormonal pathways, or if cognitive impairment itself is an expression of CNS involvement in Sjögren’s syndrome. Nevertheless, this fact is a common problem in clinical studies, as there is often no way of sufficiently differentiating the effect of those disease‐related confounders from a direct disease‐mediated effect on cognitive function. Furthermore, a standardized brain imaging was not performed in our cohort. A systematic neuroimaging approach in an outpatient setting would be beneficial for future studies.

Nevertheless, the noticeable high rate of 55% of affected patients with Neuro‐Sjögren, who suffer from cognitive impairment as well, can be derived from our data.

## Conclusion

Our results indicate an underestimation of attentional deficits in patients with Neuro‐Sjögren until now.

## Conflict of Interest

The authors declare that there is no conflict of interests regarding the publication of this paper (form included).

## Author Contributions

Conceptualization: Diana Ernst, Torsten Witte, Martin Stangel, Bruno Kopp, and Thomas Skripuletz; Formal analysis: Tabea Seeliger and Lena Jacobsen; Investigation: Tabea Seeliger, Merle Hendel, Lena Bönig, Nils Prenzler, Thea Thiele, Torsten Witte, and Bruno Kopp; Methodology: Tabea Seeliger, Lena Jacobsen, Diana Ernst, Torsten Witte, Martin Stangel, Bruno Kopp, and Thomas Skripuletz; Project administration: Tabea Seeliger and Lena Bönig; Resources: Martin Stangel; Supervision: Thomas Skripuletz; Validation: Merle Hendel and Bruno Kopp; Visualization: Tabea Seeliger; Writing – original draft: Tabea Seeliger, Lena Jacobsen, and Thomas Skripuletz; Writing – review & editing: Merle Hendel, Lena Bönig, Nils Prenzler, Thea Thiele, Diana Ernst, Torsten Witte, Martin Stangel,, and Bruno Kopp. All authors contributed to manuscript revision, read, and approved the submitted version.

## References

[1] Sjögren H . Zur Kenntnis der Keratoconjunctivitis Sicca. Acta Ophthalmol 1933;13:1–39. 10.1111/j.1755-3768.1971.tb08678.x 5171922

[2] Fox RI . Sjögren's syndrome. Lancet 2005;366:321. 16039337 10.1016/S0140-6736(05)66990-5

[3] Seeliger T , Prenzler NK , Gingele S , et al. Peripheral neuropathy with limb weakness in Sjögren’s Syndrome. Front Immunol 2019;10:1600 [cited 2019 Aug 12]. Available from: http://www.ncbi.nlm.nih.gov/pubmed/31354737 31354737 10.3389/fimmu.2019.01600 PMC6637792

[4] Tumiati B , Casoli P , Parmeggiani A . Hearing loss in the Sjögren syndrome. Ann Intern Med 1997;126:450–453. [cited 2018 Dec 21] Available from: http://www.ncbi.nlm.nih.gov/pubmed/9072930 9072930 10.7326/0003-4819-126-6-199703150-00005

[5] Delalande S , de Seze J , Fauchais A‐L , et al. Neurologic manifestations in primary Sjögren syndrome: a study of 82 patients. Medicine (Baltimore) 2004;83:280–291. [cited 2018 Dec 21]. Available from: http://www.ncbi.nlm.nih.gov/pubmed/15342972 15342972 10.1097/01.md.0000141099.53742.16

[6] Teixeira F , Moreira I , Silva AM , et al. Neurological involvement in Primary Sjögren Syndrome. Acta Reumatol Port 2013;38:29–36. (2013). [cited 2019 Apr 3]. Available from: http://www.ncbi.nlm.nih.gov/pubmed/24131909 24131909

[7] Moreira I , Teixeira F , Martins Silva A , et al. Frequent involvement of central nervous system in primary Sjögren syndrome. Rheumatol Int 2015;35:289–294. [cited 2018 Apr 23]. Available from: http://link.springer.com/10.1007/s00296‐014‐3097‐9 25056402 10.1007/s00296-014-3097-9

[8] Segal BM , Mueller BA , Zhu X , et al. Disruption of brain white matter microstructure in primary Sjögren’s syndrome: evidence from diffusion tensor imaging. Rheumatology (Oxford) 2010;49:1530–1539. [cited 2019 Aug 29]. Available from: https://academic.oup.com/rheumatology/article‐lookup/doi/10.1093/rheumatology/keq070 20444862 10.1093/rheumatology/keq070

[9] Koçer B , Tezcan ME , Batur HZ , et al. Cognition, depression, fatigue, and quality of life in primary Sjögren's syndrome: correlations. Brain Behav 2016;6:e00586 [cited 2018 Dec 21]. Available from: http://www.ncbi.nlm.nih.gov/pubmed/28032007 28032007 10.1002/brb3.586 PMC5167008

[10] Tezcan ME , Kocer EB , Haznedaroglu S , et al. Primary Sjögren’s syndrome is associated with significant cognitive dysfunction. Int J Rheum Dis 2016;19:981–988. [cited 2019 Aug 27]. Available from: http://doi.wiley.com/10.1111/1756‐185X.12912 27455357 10.1111/1756-185X.12912

[11] Morreale M , Marchione P , Giacomini P , et al. Neurological involvement in primary Sjögren syndrome: a focus on central nervous system. PLoS One 2014;9:e84605 [cited 2019 Aug 29]. Available from: https://dx.plos.org/10.1371/journal.pone.0084605 24465419 10.1371/journal.pone.0084605 PMC3896357

[12] Mataro M , Escudero D , Ariza M , et al. Magnetic resonance abnormalities associated with cognitive dysfunction in primary Sjögren Syndrome. J Neurol 2003;250:1070–1076. [cited 2019 Aug 29]. Available from: http://www.ncbi.nlm.nih.gov/pubmed/14504968 14504968 10.1007/s00415-003-0153-x

[13] Lauvsnes MB , Maroni SS , Appenzeller S , et al. Memory dysfunction in primary Sjögren's Syndrome is associated with anti‐NR2 antibodies. Arthritis Rheum 2013;65:3209–3217. [cited 2019 Aug 29]. Available from: http://www.ncbi.nlm.nih.gov/pubmed/23982950 23982950 10.1002/art.38127

[14] Takeda T , Ambrosini PJ , deBerardinis R , Elia J . What can ADHD without comorbidity teach us about comorbidity? Res Develop Disabil 2012;33:419–425. [cited 2019 Aug 29]. Available from: http://www.ncbi.nlm.nih.gov/pubmed/22119689 10.1016/j.ridd.2011.09.024 22119689

[15] Shiboski CH , Shiboski SC , Seror R , et al. 2016 American College of Rheumatology/European League Against Rheumatism classification criteria for primary Sjögren's syndrome. Ann Rheum Dis 2017;76:9–16. 27789466 10.1136/annrheumdis-2016-210571

[16] Heyman A , Fillenbaum GG , Mirra SS . Consortium to Establish a Registry for Alzheimer’s Disease (CERAD): clinical, neuropsychological, and neuropathological components. Aging Clin Exp Res 1990;2:415–424. 24.[cited 2019 Sep 10 ] Available from: http://www.ncbi.nlm.nih.gov/pubmed/2094382 10.1007/BF03323962 2094382

[17] Zimmermann P , Fimm B . Testaufmerksamkeitsbatterie zur Aufmerksamkeitsprüfung (TAP). Herzogenrath: Psytest‐Verlag, 1995.

[18] Verhülsdonk S , Hellen F , Höft B , et al. Attention and CERAD test performances in cognitively impaired elderly subjects. Acta Neurol Scandinavica. [cited 2019 Aug 12]. Available from: http://www.ncbi.nlm.nih.gov/pubmed/25352352 10.1111/ane.12346 25352352

[19] Schmid NS , Ehrensperger MM , Berres M , et al. The extension of the German CERAD neuropsychological assessment battery with tests assessing subcortical, executive and frontal functions improves accuracy in dementia diagnosis. Dement Geriatr Cogn Disord Extra 2014;4:322–334 [cited 2018 Dec 21]. Available from: https://www.karger.com/Article/FullText/357774 10.1159/000357774 PMC4176468 25298776

[20] Kopp B , Steinke A , Bertram M , et al. Multiple levels of control processes for wisconsin card sorts: an observational study. Brain Sci 2019;9:141. 10.3390/brainsci9060141 PMC6627185 31213007

[21] Zimmermann P , Fimm B . Testbatterie zur Aufmerksamkeitsprüfung (TAP) Version 1.02c – Handbuch Teil 2 (Statistiken). Herzogenrath: Psytest, 1994.

[22] Schulz D , Kopp B , Kunkel A , Faiss JH . Cognition in the early stage of multiple sclerosis. J Neurol 2006;253:1002–1010. [cited 2020 Jan 9]. Available from: http://www.ncbi.nlm.nih.gov/pubmed/16609812 16609812 10.1007/s00415-006-0145-8

[23] Chisholm DM , Waterhouse JP , Mason DK . Lymphocytic sialadenitis in the major and minor glands: a correlation in postmortem subjects. 1970 [cited 2018 Oct 26]. Available from: https://www‐1ncbi‐1nlm‐1nih‐1gov‐1mf9loa951625.han.mh‐hannover.de/pmc/articles/PMC476869/pdf/jclinpath00088‐0042.pdf 10.1136/jcp.23.8.690 PMC476869 5488040

[24] Lafitte C , Amoura Z , Cacoub P , et al. Neurological complications of primary Sjögren’s syndrome. J Neurol 2001;248:577–584. [cited 2019 Aug 29]. Available from: http://www.ncbi.nlm.nih.gov/pubmed/11517999 11517999 10.1007/s004150170135

[25] Le Guern V , Belin C , Henegar C , et al. Cognitive function and 99mTc‐ECD brain SPECT are significantly correlated in patients with primary Sjogren syndrome: a case‐control study. Ann Rheum Dis 2010;69:132–137.[cited 2019 Aug 29]. Available from: http://ard.bmj.com/cgi/doi/10.1136/ard.2008.090811 19158115 10.1136/ard.2008.090811

[26] Indart S , Hugon J , Guillausseau PJ , et al. Impact of pain on cognitive functions in primary Sjögren syndrome with small fiber neuropathy. Medicine (Baltimore) 2017;96:e6384 [cited 2019 Aug 29]. Available from: http://insights.ovid.com/crossref?an=00005792‐201704210‐00010 28422829 10.1097/MD.0000000000006384 PMC5406045

[27] Martínez S , Cáceres C , Mataró M , et al. Is there progressive cognitive dysfunction in Sjögren Syndrome? A preliminary study. Acta Neurol Scand 2010;122:182–188. [cited 2019 Aug 29]. Available from: http://doi.wiley.com/10.1111/j.1600‐0404.2009.01293.x 20096020 10.1111/j.1600-0404.2009.01293.x

[28] Segal BM , Pogatchnik B , Holker E , et al. Primary Sjogren’s syndrome: cognitive symptoms, mood, and cognitive performance. Acta Neurol Scand 2012;125:272–278. [cited 2019 Aug 29]. Available from: http://www.ncbi.nlm.nih.gov/pubmed/21651503 21651503 10.1111/j.1600-0404.2011.01530.x PMC3188671

[29] Harboe E , Tjensvoll AB , Maroni S , et al. Neuropsychiatric syndromes in patients with systemic lupus erythematosus and primary Sjögren syndrome: a comparative population‐based study. Ann Rheum Dis 2009;68:1541–1546. [cited 2019 Aug 12]. Available from: http://ard.bmj.com/cgi/doi/10.1136/ard.2008.098301 18930990 10.1136/ard.2008.098301

[30] Epstein LC , Masse G , Harmatz JS , et al. Characterization of cognitive dysfunction in Sjögren’s syndrome patients. Clin Rheumatol 2014;33:511–521. [cited 2019 Aug 29]. Available from: http://link.springer.com/10.1007/s10067‐013‐2453‐6 24337727 10.1007/s10067-013-2453-6

[31] Rodrigues D‐N , Hora JSI , Salgado MCF , et al. A short neuropsychological evaluation of patients with primary Sjögren’s syndrome. Arq Neuropsiquiatr 2014;72:38–43. [cited 2019 Aug 29]. Available from: http://www.scielo.br/scielo.php?script=sci_arttext&pid=S0004‐282X2014000100038&lng=en&tlng=en 24637981 10.1590/0004-282X20130195

